# Histone Deacetylase Inhibitors Facilitate Dihydroartemisinin-Induced Apoptosis in Liver Cancer *In Vitro* and *In Vivo*

**DOI:** 10.1371/journal.pone.0039870

**Published:** 2012-06-28

**Authors:** Chris Zhiyi Zhang, Yinghua Pan, Yun Cao, Paul B. S. Lai, Lili Liu, George Gong Chen, Jingping Yun

**Affiliations:** 1 State Key Laboratory of Oncology in Southern China, Sun Yat-Sen University Cancer Center, Guangzhou, China; 2 Department of Pathology, Sun Yat-Sen University Cancer Center, Guangzhou, China; 3 Department of Rheumatology and Immunology, The Third Affiliated Hospital of Sun Yat-Sen University, Guangzhou, China; 4 Department of Surgery, Prince of Wales Hospital, The Chinese University of Hong Kong, Shatin, N.T., Hong Kong; Vanderbilt University Medical Center, United States of America

## Abstract

Liver cancer ranks in prevalence and mortality among top five cancers worldwide. Accumulating interests have been focused in developing new strategies for liver cancer treatment. We have previously showed that dihydroartemisinin (DHA) exhibited antitumor activity towards liver cancer. In this study, we demonstrated that histone deacetylase inhibitors (HDACi) significantly augmented the antineoplastic effect of DHA via increasing apoptosis *in vitro* and *in vivo*. Inhibition of ERK phosphorylation contributed to DHA-induced apoptosis, due to the fact that inhibitor of ERK phosphorylation (PD98059) increased DHA-induced apoptosis. Compared with DHA alone, the combined treatment with DHA and HDACi reduced mitochondria membrane potential, released cytochrome *c* into cytoplasm, increased p53 and Bak, decreased Mcl-1 and p-ERK, activated caspase 3 and PARP, and induced apoptotic cells. Furthermore, we showed that HDACi pretreatment facilitated DHA-induced apoptosis. In Hep G2-xenograft carrying nude mice, the intraperitoneal injection of DHA and SAHA resulted in significant inhibition of xenograft tumors. Results of TUNEL and H&E staining showed more apoptosis induced by combined treatment. Immunohistochemistry data revealed the activation of PARP, and the decrease of Ki-67, p-ERK and Mcl-1. Taken together, our data suggest that the combination of HDACi and DHA offers an antitumor effect on liver cancer, and this combination treatment should be considered as a promising strategy for chemotherapy.

## Introduction

Liver cancer is the fifth most common cancer worldwide and the third most common cause of death from cancer [1]. More than 75% of new cases are diagnosed in developing countries; however, incidence is increasing in economically developed regions, including Japan, Western Europe, and the United States [2], [3]. Although surgical resection and liver transplant are the two major therapeutic options with curative potential, surgery is only feasible for about 20% of liver cancer cases since patients are most often diagnosed at an advanced stage [4], [5]. To date, chemotherapy for liver cancer is not satisfactory and the long-term survival of liver cancer patients is still poor [4], [6]. Therefore, developing novel and effective therapeutic strategies for liver cancer is of great need and significance.

Histone deacetylase inhibitors (HDACi) are currently a major focus of interest as antineoplastic agents [7], [8]. HDACi is a class of agents that function via blocking histone deacetylation, thereby modifying chromatin structure and gene transcription [9]. Particularly, HDACi inhibit the acetylation of lysine residues at the histone N-terminal tail which results in loosening the association of histones with DNA, thereby allowing the expression of genes related to tumor suppression [10]. Understanding the association between HDAC activities and various cancers led many researchers to consider HDAC inhibitors as potent agents that can interfere with cancer cell proliferation and/or survival through the modulation of cell cycle progression, differentiation, or by promoting cell death. For example, Kim et al. reported that CG0006 exposure in breast cancer cell resulted in cell death via down-regulation HDAC6 [11]. Bommi et al. demonstrated that sodium butyrate induced apoptosis in cancer cells by transcriptional downregulation of BMI1 [12].

Although HDACi alone may be clinically useful, they will most likely be of value in combination with other antitumor agents. SAHA has been approved by the U.S. Food and Drug Administration (FDA) for the treatment of cutaneous T cell lymphoma and other HDACi are now undergoing Phase I/II clinical trials as a single agent or in combination with other agents [13], [14]. Accumulating reports have been indicated the synergistic effect on lethality of combination of HDACi and other chemotherapeutic agents. Kretzner et al. showed that combination of HDACi and Aki enhanced lymphoma cell death through repression of c-Myc, hTERT, and microRNA levels [15]. Nguyen et al. reported that coadministration of HDACi synergistically increased KW-2449 lethality resulting from inactivation of Bcr/Abl [16]. Lately, a phase II study revealed that treatment of vorinostat combined with tamoxifen significantly prolonged the survival of patients with breast cancer [17]. However, such a synergistic effect has rarely been demonstrated in liver cancer.

Recently, we have reported that Dihydroartemisinin (DHA), the main active metabolite of artemisinin derivatives, exhibited anticancer activity towards liver cancer [18]. In the present study, we showed that (a) DHA induced apoptosis via downregulating ERK phosphorylation, which was further confirmed by the data that the inhibitor of ERK phosphorylation (PD98059) increased DHA-induced apoptosis, (b) HDACi *in vitro* remarkably enhanced DHA-induced cell death, accompanying with reduction of mitochondria membrane potential, release of cytochrome *c* into cytoplasm, increase of p53 and Bak, and decreases of Mcl-1 and p-ERK, (c) the combination of HDACi and DHA *in vivo* significantly halted the growth of liver cancer tumor xenograft. Our data may suggest the combination of HDACi and DHA as a promising strategy for liver cancer chemotherapy.

## Materials and Methods

### Cell culture

Human liver cancer cell lines (Hep G2 and PLC/PRF/5) were purchased from American Type Culture Collection (ATCC, Manassas, VA) and cultured in Dulbecco's modified Eagle's medium (DMEM) (Gibco, Gaithersburg, MD) containing 10% fetal bovine serum (FBS), 100 mg/ml penicillin, and 100 mg/ml streptomycin in a humidified atmosphere of 5% CO_2_ and 95% air at 37°C.

### Antibodies and reagents

Antibodies for Mcl-1, PARP, Bak, and Actin were purchased from Santa Cruz Biotechnology (Santa Cruz, CA). Antibodies for caspase 3, p38, p-p38, ERK, p-ERK, JNK, and p-JNK were provided by Cell Signaling (Danvers, MA). Dihydroartemisinin (DHA, dissolved in DMSO), sodium butyrate (NaB, dissolved in H_2_O), suberoylanilide hydroxamic acid (SAHA, dissolved in DMSO) and p-ERK inhibitor (PD98059, dissolved in DMSO) were purchased from Sigma (St. Louis, MO).

### MTT

Cell viability was assessed by 3-(4, 5-dimethylthiazol-2-yl)-2, 5-diphenyltetrazo-lium bromide (MTT) assay. Briefly, 8×10^3^ of cells were seeded into 96-well plates for 24 h, followed by incubation with various doses of DHA for indicated time. After adding 100 μl/well of MTT solution, the cells were incubated for another 2 h. Supernatants were then removed and the formazan crystals were dissolved in 100 μl/well DMSO. The absorbance at 570/630 nm of each sample was measured using multilabel plate reader (PerkinElmer). Three independent experiments were performed.

### Colony formation

One hundred of cells were seeded into 6-well plates, and cultured for 7 d. And then the medium was replaced by fresh one containing DHA. After being incubated for another 7 d, colony formed by liver cancer cells was stained with 0.05% crystal violet (Sigma, St. Louis, MO) for 8 min. The number of colony was then quantified.

### Western blot

Cell lysates were boiled with 6x sodium dodecyl sulfate (SDS) loading buffer and then fractionated by SDS-PAGE. The proteins were transferred to PVDF membrane which was then incubated with a primary specific antibody in 5% of non-fat milk, followed by a horse radish peroxidase (HRP)-conjugated anti-mouse or anti-rabbit second antibodies. ECL detection reagent (Amersham Life Science, Piscataway, NJ) was used to demonstrate the results.

### Annexin V/PI assay

Apoptosis was assessed using Annexin V-PI double staining. After treatments, cells were trypsinized, and stained with 0.5 mg/ml Annexin V in binding buffer (10 mM HEPES free acid, 0.14 M NaCl, and 2.5 mM CaCl_2_) for 30 min. Afterward, PI (5 μg/mL final concentration) was added and incubated for another 15 min. Cells were applied to a flow cytometer for data collection.

### TUNEL assay

Apoptosis assay was performed using Apo-Direct TUNEL Assay kit (Millipore). Cells were harvested and fixed in 4% PFA for 60 min at 4°C, followed by a second fixation in 70% (v/v) ethanol overnight at −20°C. Cells were then treated with various reagents for a designed period according to the manufacture's instruction. Finally, cells were analyzed by flow cytometry using FACS Vantage machine (Becton Dickinson). The Cell Quest software (Verity Software House) was used to analyze the data.

### *In situ* cell death detection

Labeling of fragmented DNA to assess apoptosis was performed with TUNEL staining (green fluorescence), using *In Situ* Cell Death Detection Kit (Roche, LA), as described in our previous study [19].

### Measurement of caspase 3 activity

The activity of caspase 3 induced by DHA treatment was determined by the Caspase-3 Activity Assay Kit (Merck, Darmstadt).

### Measurement of mitochondrial membrane potential (Δψ_m_) by flow cytometry

Forty nM of DioC6 (Sigma–Aldrich, MO) were incubated with treated cells at indicated time points for 15 min at 37°C. The harvested cells were washed with ice-cold PBS and analyzed by flow cytometry using Becton Dickinson FACS Vantage machine (Becton Dickinson, NJ). Cells with low Δψ_m_ were presented as a percentage of the total cell population. The CellQuest software (Verity Software House) was used to analyze the data.

### Animal studies

All animal experiments were conducted according to relevant national and international guidelines and have been approved by the Institute Research Medical Ethics Committee of Sun Yat-Sen University Cancer Center. 1×10^7^ of Hep G2 cells were suspended in sterile PBS and injected subcutaneously into the right flank of the mice. Mice were checked daily for xenograft/tumor development. Mice were randomized into three groups of 6 mice/ group. DHA (5 mg/kg mouse body weight) was given to the ‘DHA’ group, SAHA (1.5 mg/kg mouse body weight) was given to the ‘SAHA’ group, combination of SAHA and DHA was given to ‘DHA+SAHA’ group, once daily for five consecutive days per week for 24 d. The DMSO group received an equal volume of solvent control. After treatment at various time intervals, mouse body weight and tumor size were measured. Finally, tumors were excised, weighed and fixed in 4% of PFA. Paraffin-embedded tissues were then sectioned at 4 nm and ready for H&E staining and TUNEL assay.

### Immunohistochemistry

Formalin-fixed and paraffin-embedded liver cancer sections with a thickness of 4 μm were dewaxed in xylene and graded alcohols, hydrated, and washed in phosphatebuffered saline (PBS). After pretreatment in a microwave oven, endogenous peroxidase was inhibited by 3% hydrogen peroxide in methanol for 20 min, followed by avidin-biotin blocking using a biotin-blocking kit (DAKO, Germany). Slides were then incubated with antibodies for 4 h in a moist chamber at room temperature, washed in PBS, and incubated with biotinylated goat anti-rabbit/mouse antibodies. Slides were developed with the Dako Liquid 3, '3-diaminobenzidine tetrahydrochloride (DAB) +Substrate Chromogen System and counterstained with hematoxylin.

### Statistical analysis

Difference between groups was determined for statistical significance using one-way ANOVA or Student's *t*-test. All *P*-values are two-sided and *P*<0.05 was considered as statistically significant. All statistical calculations were performed with the SPSS software (SPSS, Inc., Chicago, IL). The data were presented as mean±SD from at least three independent experiments.

## Results

### Activations of MAP kinases were involved in DHA-induced apoptosis

**Figure 1:**
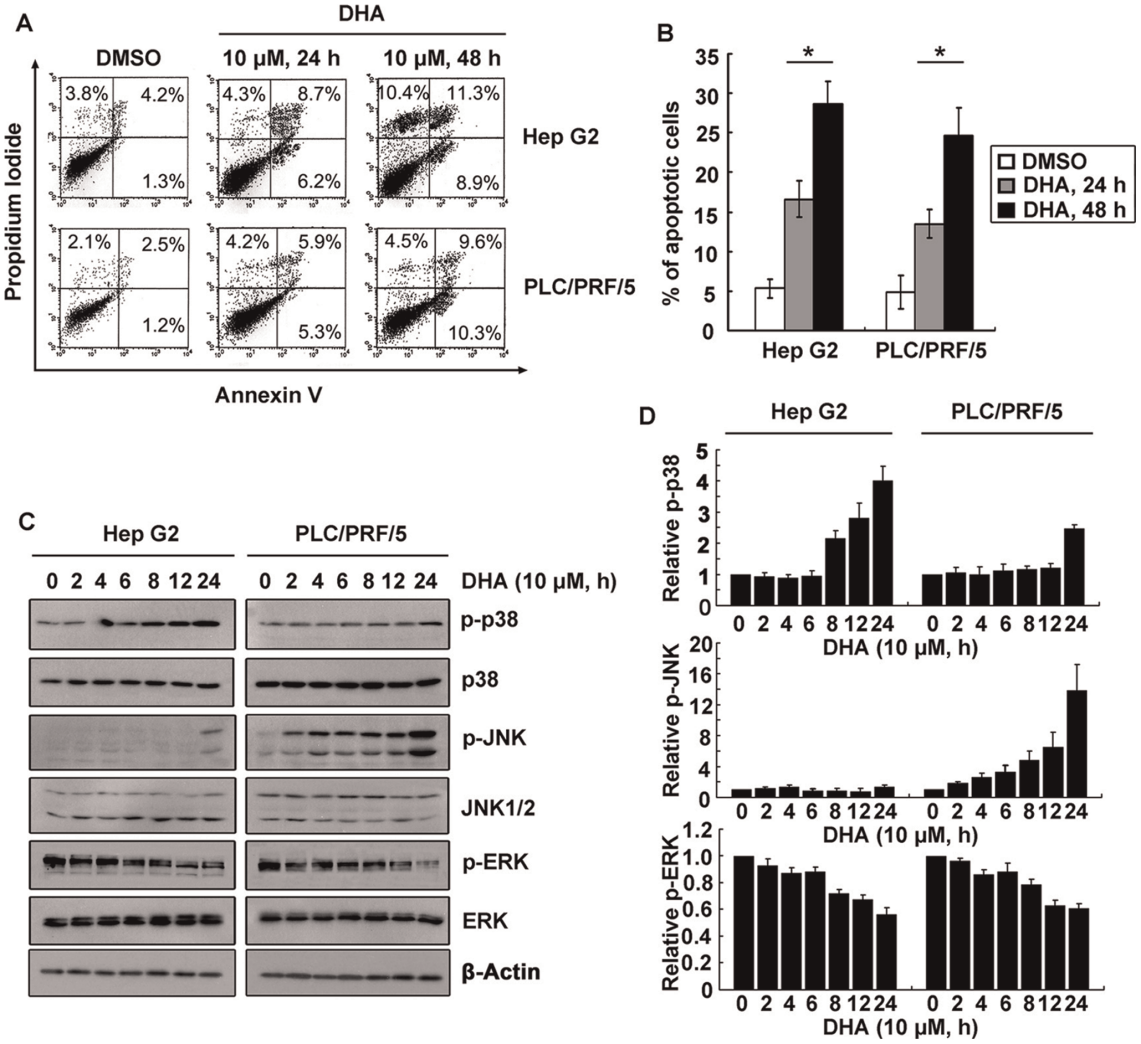
Activations of MAP kinases were involved in DHA-induced apoptosis. **A.** DHA induced apoptosis in liver cancer cells. Cells treated with either DMSO or 10 μM DHA for 24 and 48 h were stained with both Annexin V and Propidium Iodide (PI) for 45 min. Apoptosis induced by DHA was then assessed by flow cytometer analysis. **B.** The percentage of apoptotic cells were shown, after quantitative analysis of PI/Annexin V assay. Data are presented as mean±SD of three independent experiments. *P<0.05, versus the DMSO group. **C.** MAP kinases were activated by DHA. Cells were treated with 10 μM DHA for indicated time. The phosphorylation of p38, ERK and JNK was determined. **D.** Quantitative data from three independent experiments were shown to indicate the relative expression of p-p38, p-JNK, and p-ERK.

DHA has been demonstrated to induce cell death in human cancers [20], [21]. We first assessed DHA-induced apoptosis in liver cancer cell lines, using Annexin V assay. Results indicated that percentage of Annexin V-positive cells were dramatically increased upon DHA treatment (Fig. 1A), suggesting DHA being a potent apoptosis inducer in liver cancer cells. Compared to the control, following exposure of 10 μM DHA for 24 h, the percentage of apoptotic cells was remarkably increased from 5.3% and 4.9% to 16.6% and 13.5%, respectively in Hep G2 and PLC/PRF/5 cells (Fig. 1B).

Apoptosis induction usually associates with activation of MAP kinases. A time-course analysis was performed on the phosphorylation levels of three MAP kinase members, including extracellular signal-regulated kinase (ERK), c-Jun NH2-terminal kinase (JNK) and p38 (Fig. 1C). The protein levels of all 3 MAP kinases remained unchanged. However, p38 phosphorylation was increased after DHA treatment in both tested cells. The level of JNK phosphorylation remained the same as control in Hep G2 cells, but was markedly increased in PLC/PRF/5 cells (Fig. 1D). The levels of p-ERK appeared decreasing in both liver cancer cells treated with DHA. These data may suggest that inactivation of ERK contributes to DHA-induced apoptosis.

### Inhibition of ERK phosphorylation was attributed to DHA-induced apoptosis in liver cancer cells

**Figure 2:**
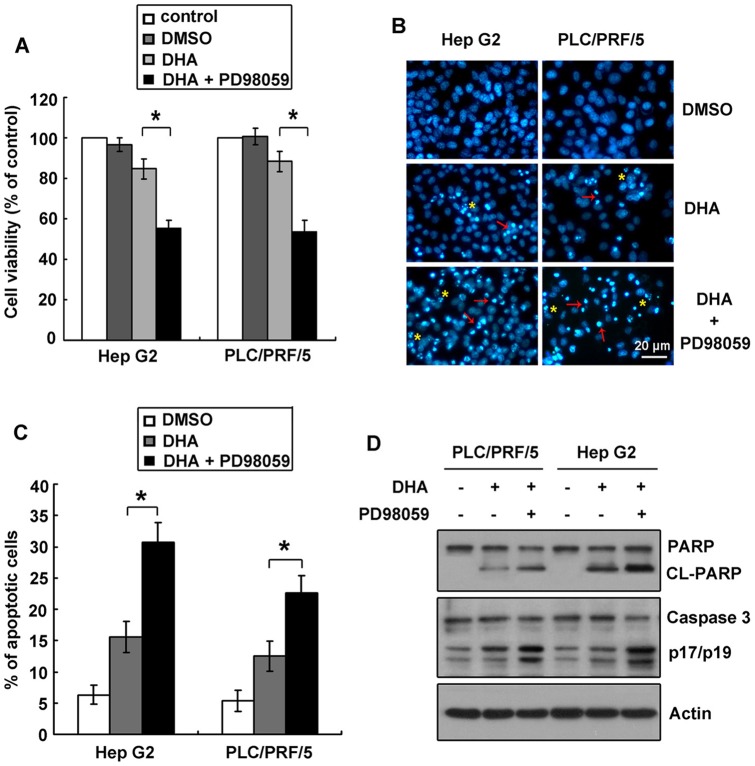
Inhibition of ERK phosphorylation contributed to DHA-induced apoptosis. A. PD98059, an inhibitor of ERK phosphorylation, enhanced DHA-induced decrease of cell viability. Cells were pretreated with 10 μM PD98059 for 2 h, and then incubated with 10 μM DHA for another 24 h. The residual cell viability was determined by MTT assay. Data are mean ± SD of three independent experiments, *P<0.05. **B.** PD98059 treatment increased the production of apoptotic body. Cells pretreated with 10 μM PD98059 for 2 h, and then incubated with 10 μM DHA for another 24 h were stained with Hoechst 33342 dye. DNA fragmentation (indicated by asterisks) and nuclear condensation (denoted by arrows) were observed under a fluorescence microscope. **C.** PD98059 treatment enhanced DHA-induced apoptosis. TUNEL assay was performed to determine apoptosis. The number of apoptotic cells was determined and the percentage was indicated by histogram. *P<0.05. **D.** PD98059 plus DHA treatment led to cleavages of PARP and caspase 3. Proteins collected from cells treated with PD98059 and DHA for 24 h were subjected to western blot to detect the cleavages of PARP and caspase 3. Actin was used as a loading control.

To test our assumption that inactivation of ERK was involved in DHA-induced apoptosis, we pretreated cells with PD98059, an inhibitor of ERK phosphorylation. Firstly, the cytotoxicity of PD98059 was tested. Results indicated that PD98059 alone did not cause significant apoptosis in both cells (data not shown). We next determined the effect of PD98059 on DHA-induced cell growth attenuation. As indicated by MTT result, PD98059 significantly reduced liver cancer cell viabilities, compared with DHA groups (Fig. 2A).

Next, we examined whether PD98059 treatment enhanced DHA-induced cell growth inhibition through inducing apoptosis. We assessed DHA-induced apoptosis in liver cancer cells pretreated with 10 μM PD98059 for 2 h by Hoechst 33342 staining. Results revealed more cells with characteristic features including chromatin condensation and apoptotic body presented in PD98059-pretreated cells (Fig. 2B). This was further confirmed by TUNEL assays showing that the percentages of TUNEL-positive cells were increased in liver cancer cells treated with both PD98059 and DHA (Fig. 2C). Moreover, levels of cleaved PARP and cleaved caspase 3 were noticeably increased by the ERK inhibitor in the 2 liver cancer cell lines (Fig. 2D). These findings suggest that DHA-induced apoptosis may be related to ERK phosphorylation.

### HDACi facilitated DHA-induced apoptosis in liver cancer cells

**Figure 3:**
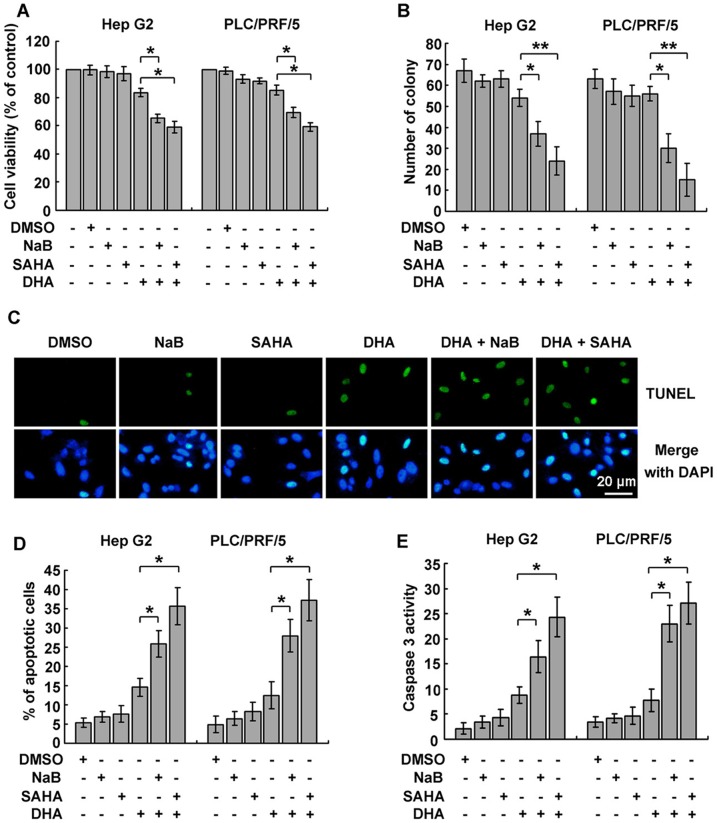
HDACi facilitated DHA-induced apoptosis. A. Combination treatment with HDACi and DHA increased cell death. Cells were treated with 4 mM NaB, 1.25 μM SAHA, 10 μM DHA or combination of NaB/SAHA and DHA for 24 h. Cell viabilities were measured by MTT assay. **B.** The inhibitory effect of HDACi and DHA in combination on liver cancer cell growth was further confirmed by colony formation assay. One hundred of cells were seeded into 6-well plates for 7 d, and then cultured with either HDACi or DHA for another 7 d. Colonies were stained with 0.05% crystal violet. The number of colony in each well was counted and statistical analysis was performed. Data are presented as mean ± SD of three independent experiments. **C.** The effect of HDACi on DHA-induced apoptosis was measured by TUNEL assay, using *in situ* cell death detection kit. Hep G2 cells treated as described in **A** were subjected to TUNEL assay. Apoptotic cells were observed under fluorescent microscope. **D.** The effect of HDACi and DHA in combination was further confirmed by TUNEL assay, using flow cytometry. Percentage of apoptotic cells was calculated. **E.** Caspase 3 activation was involved in HDACi-mediated apoptosis in cells treated with DHA. The activity of caspase 3 in cells treated as described in **A** was determined and the relevant change was shown. For **A**, **B**, **D** and **E**, *P<0.05, **P<0.01, versus the DHA group.

In view of that HDACi is capable of enhancing the lethal effect of chemotherapeutic agents [22], [23], we intended to examine whether DHA combined with HDACi resulted in more cell death in liver cancer. NaB and SAHA were used in MTT analysis. According to the results, combination of DHA and HDACi significantly reduced cell viabilities in liver cancer cells, compared to treatment with single agent (Fig. 3A). The increased cytotoxicity of combination of DHA and HDACi was also determined by colony formation assay. Cells in DMSO groups formed a number of visible colonies in 15 d. The number of colony formed by cells cultured with both DHA and HDACi was significantly less than that with DHA alone (Fig. 3B).

Next we determined the pro-apoptotic activity of combined treatment with DHA and HDACi. DHA treatment potently induced apoptosis in liver cancer cells, but more apoptosis was induced by the combined treatment with both agents, as shown by TUNEL assays indicating a noticeable increase in TUNEL-positive cells (Fig. 3C). Statistically, DHA in combination with NaB or SAHA increased apoptotic cells by 1.9 or 2.8 fold, respectively in Hep G2 cells, and by 3.0 or 3.5 fold respectively in PLC/PRF/5 cells (Fig. 3D). In line with the increased apoptosis, caspase 3 activity was higher in cells treated with both DHA and HDACi (Fig. 3E).

### Release of cytochrome *c* into cytoplasm and downregulation of Mcl-1 and p-ERK contributed to apoptosis caused by the combined treatment with HDACi and DHA

**Figure 4:**
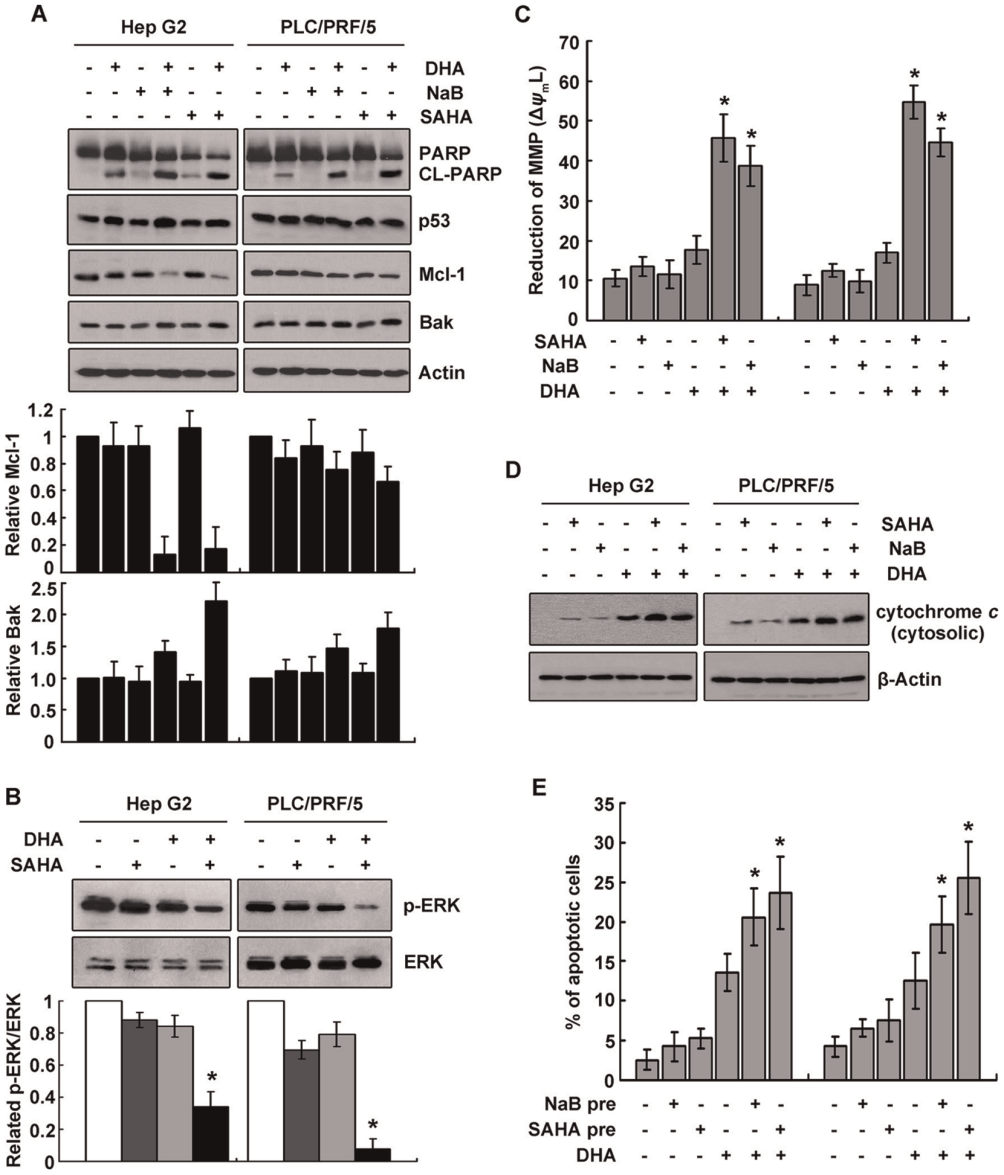
HDACi exposure in DHA-treated cells enhanced decreases of Mcl-1 and p-ERK. A. Combination treatment with HDACi and DHA resulted in downregulation of Mcl-1 and upregulation of Bak. liver cancer cells were exposed to 4 mM NaB, 1.25 μM SAHA, 10 μM DHA or combination of NaB/SAHA and DHA for 24 h. Expression of Mcl-1, Bak and cleaved PARP was examined by western blot. Upper panel: a representative result was shown. Bottom panel: the relative expression of Mcl-1 and Bak normalized to Actin was indicated by histogram. **B.** The expression of p-ERK was reduced in cells treated with HDACi and DHA. Proteins collected from liver cancer cells treated with SAHA, DHA or the combined drugs were subjected to western blot to examine p-ERK expression. Upper panel: a representative result was presented. Bottom panel: the relative expression of p-ERK/ERK was shown. **C.** Reduction of mitochondrial membrane potential (MMP) was induced in HDACi/DHA-treated cells. Hep G2 and PLC/PRF/5 cells were treated as described in **A**. The MMP collapse (Δψ_m_L) was measured by flow cytometry after staining the cells with DioC6 and quantitative analysis of Δψ_m_ was shown. The data represented mean±SD of three independent experiments. **D.** Cytochrome *c* was released to cytosol in treated cells. Cells were treated as described in **A**. Fractions of cytosol were isolated to examine the distribution of cytochrome *c*. β-Actin was used as the marker for cytosol. **E.** HDACi pretreatment sensitized cells to DHA-induced apoptosis. liver cancer cells pretreated with 4 mM NaB or 1.25 μM SAHA for 2 h were further exposed to 10 μM DHA for another 24 h. TUNEL assays were performed to determine apoptosis. The percentage of TUNEL-positive cells was shown. For **B**, **C** and **E**, *P<0.05, versus the DHA group.

We have previously demonstrated that DHA-induced apoptosis associated with Mcl-1 degradation and Bak activation [18]. We next examined whether Mcl-1 and Bak were involved in HDACi-mediated enrichment of apoptosis in DHA-treated cells. As indicated in results of western blot, Mcl-1 was dramatically decreased, whereas Bak was markedly increased in Hep G2 cells treated with both DHA and NaB/SAHA. The alterations of Mcl-1 and Bak in PLC/PRF/5 cells shared a similar trend with those in Hep G2 cells (Fig. 4A). In addition, wild-type p53 in Hep G2 cells were upregulated, while mutant p53 in PLC/PRF/5 cells were hardly affected, following the treatment (Fig. 4A).

In light of emerging data that ERK phosphorylation was inhibited in DHA-treated and HDACi-treated cells. We next examined the level of phosphorylated ERK. Results showed a rapid decrease of p-ERK in liver cancer cells treated with both DHA and SAHA, especially in PLC/PRF/5 cells (Fig. 4B).

Since DHA-induced apoptosis was attributed to the depolarization of mitochondrial outer membrane [18], we next examined the reduction of mitochondrial membrane potential (MMP). Results showed that the combined treatment remarkably lowered the mitochondrial transmembrane potential (Fig. 4C), followed by an obvious release of cytochrome *c* from mitochondria to cytoplasm (Fig. 4D).

As shown in our previous study, HDACi pretreatment sensitized liver cancer cells to etoposide [22]. We pretreated cells with NaB or SAHA, and then assessed the resulting apoptosis by TUNEL assays. Compared to those of unpretreated cells, percentages of TUNEL-positive cells in HDACi-pretreated cells were significantly increased (Fig. 4E).

### SAHA enhanced antitumor effect of DHA on Hep G2 xenograft tumor in mice

**Figure 5:**
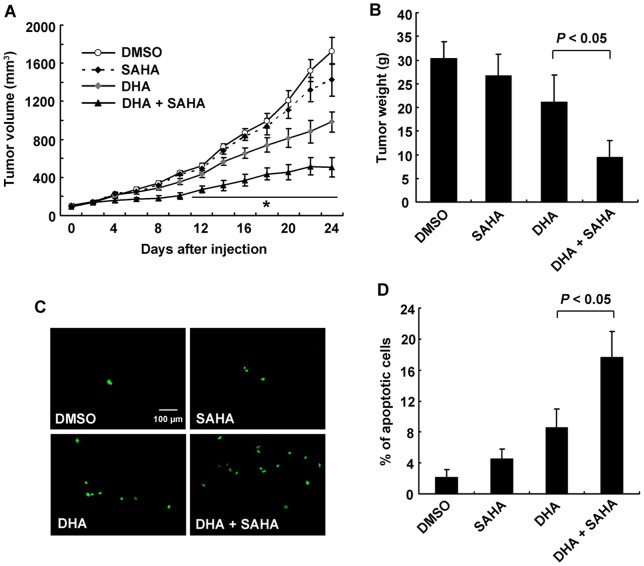
SAHA enhanced antitumor effect of DHA on Hep G2 xenograft tumor in mice. A. Combination of SAHA and DHA noticeably halted the growth of Hep G2 xenograft tumor. Nude mice were inoculated with 1×10^7^ of Hep G2 cells. After the formed tumor was palpable, mice were randomly divided into four groups. DHA (5 mg/kg mouse body weight) was given to the ‘DHA’ group, SAHA (1 mg/kg mouse body weight) was given to the ‘SAHA’ group, combination of SAHA and DHA was given to 'DHA+SAHA' group, once daily for five consecutive days per week for 24 d. The tumor volumes were calculated every two days. Six xenografts were performed in each group. Data are mean±SD, *P<0.05, versus the DMSO group. **B.** Combination of SAHA and DHA treatments resulted in a dramatic decline of tumor weight. On day 24, mice were sacrificed, and the tumor weights were measured. **C.** Apoptosis was induced *in vivo*. Tumors were sectioned and apoptosis was determined using *in situ* cell death detection kit. Apoptotic cells were observed under fluorescent microscope. **D.** SAHA significantly increased apoptosis in DHA-treated mice. Percentages of apoptotic cells were measured by counting the number of green cells under five random fields.

Having demonstrated the ability of SAHA to enhance DHA-mediated cell death *in vitro*, we further determined the synergetic effect of SAHA and DHA *in vivo*. Hep G2 cells were subcutaneously injected in nude mice to establish tumor xenograft. Nude mice bearing tumor xenografts were dosed with DHA (5 mg/kg/Bid) and/or SAHA (1.5 mg/kg/Bid) daily for 24 days. The treatment did not appear to have a noticeable effect on body weight in mice. On average, the combination therapy inhibited liver cancer tumor growth by more than 44.7% while the single agent treatment with either DHA or SAHA only inhibited the tumor growth by 17.6% and 4.6%, respectively (Fig. 5A). On Day 24, mice were sacrificed and the tumor weights were measured. As expected, the combination of HDACi and DHA significantly reduced the weights of xenograft tumor, compared with DHA-only groups (Fig. 5B). These data indicated that the combination treatment generated a greater anti-proliferative effect and cytotoxicity than either single agent alone in liver cancer xenografts *in vivo*.

**Figure 6:**
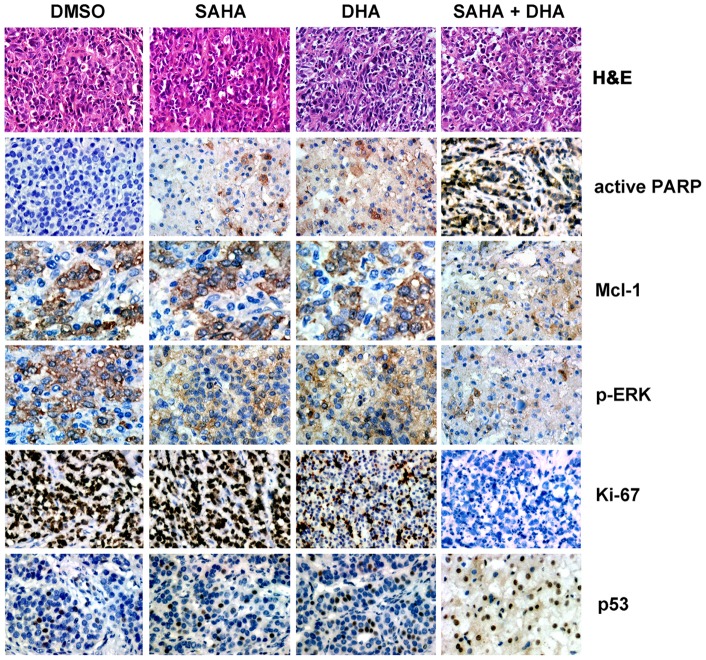
Decreased expression of Mcl-1 and increased levels of active PARP and cleaved caspase 3 were recorded in HDACi/DHA-treated mice. SAHA enlarged the apoptotic region caused by DHA treatment in Hep G2 xenograft tumor. Tumors were excised and subjected to H&E staining for determination of pathological evaluation. On the other hand, tissues of xenografts were subjected to immunochemistry to detect the expression of Ki-67, p53, Mcl-1, p-ERK, and active PARP. Original magnification ×400.

In order to test whether HDACi enhanced the lethal effect of DHA via increasing apoptosis, tumor tissues were sectioned and subjected to *in situ* cell death detection (Fig. 5C). Results showed that the proportion of apoptotic cells was significantly increased from 8.6±2.4% in DHA group to 17.7±3.3% in combined treatment group (Fig. 5D). In addition, we examined the histology of tumors after the treatment using H&E staining. Tumors from control group showed typical histological appearance of liver cancer (Fig. 6). The sections of DHA-treated tumors showed that cancer cells were markedly decreased, with signs of apoptosis, infiltration of inflammatory cells and fibrosis. In the combined treated group, apoptotic regions and extensive necrosis with infiltration of phagocytic cells could be observed fairly often.

In addition, we performed immunohistochemistry to detect the proteins involved in HDACi/DHA-induced apoptosis (Fig. 6). Decreased expression of Ki-67 indicated the reduction of cell proliferation and likely enhanced cell death. Detectable difference in p53 expression was observed. Striking increase of active PARP, as well as a predominant decline of Mcl-1 and p-ERK, was present in HDACi/DHA-treated xenograft. Taken together, these data indicated that HDACi were able to significantly augment DHA-mediated antitumor effects.

## Discussion

Recent studies suggest that HDACi including NaB and SAHA interact synergistically with cytotoxic agents, such as fludarabine and etoposide, to dramatically increase mitochondrial injury and apoptosis in leukemic and epithelial cancer cells [24], [25]. The antitumorigenic properties of HDACi are especially notable probably due to the fact that their cytotoxic effects are usually specific to cancer cells but not to normal cells. However, when used as a single agent, HDACi might exhibit limited lethal activity towards liver cancer, which is evident in the present study showing that both NaB and SAHA at low doses are unable to induce significant growth inhibition *in vitro* and *in vivo*. However, when HDACi are used in combination with DHA, a derivative of artemisinin that is clinically used in malaria treatment with good toxicity profile [26], they can induce much more apoptosis, resulting in remarkable halt of tumor xenograft in nude mice. There is very limited information on HDACi in combination with other anti-tumor agents against liver cancer. Our data for the first time have demonstrated a synergic effect of DHA and HDACi in inhibition of liver cancer.

Many reports have demonstrated that the threshold of apoptosis in cancer cells can be controlled by the activities of multiple signal transduction pathways, one of which is Raf-MEK1/2-ERK1/2 pathway [27], [28]. This pathway is frequently dysregulated in neoplastic transformation, along with the c-Jun NH2-terminal kinase (JNK1/2) and p38 MAPK pathways [29]. It has also been implicated that activation of the ERK1/2 pathway is usually associated with survival but JNK1/2 and p38 MAPK pathway with apoptosis [30]. In our study, ERK1/2 phosphorylation was slightly inhibited by DHA treatment but strongly inhibited by the combined treatment with DHA and HDACi. In addition, using the ERK-specific inhibitor PD98059, we demonstrated that the activation of the ERK is antiapoptotic since the ERK inhibitor enhanced DHA-induced apoptosis in liver cancer cells.

A number of antiapoptotic effector proteins have been identified downstream of ERK1/2 signaling, including Bcl-xL and Mcl-1 [31], [32]. Alterations of both phosphorylated ERK and Mcl-1 frequently occurs in the same direction. Yuen et al. reported that silencing Ran may lead to deactivation of ERK and downregulation of Mcl-1 in cancer cells [33]. Reeves et al. showed that the activation of ERK and induction of Mcl-1 were observed in myeloid cells infected by human cytomegalovirus [34]. Calviño et al. reported treatement with lonidamine plus arsenic trioxide resulted in reductions of Mcl-1 and p-ERK [35]. In our previous study, Mcl-1 was downregulated in DHA-treated cells [ref]. Here we further showed a decrease of phosphorylated ERK in DHA-exposed liver cancer cells. Collectively, it seems that there is some certain correction between Mcl-1 and p-ERK: one protein could be regulated by the other. Interestingly, Konopleva et al. showed that MEK inhibitors such as PD0325901 and CI-1040, which are capable of inhibiting the activation of ERK, successfully suppressed Mcl-1 expression in Leukemia cells [36]. Booy et al. demonstrated that knockdown of ERK1 or inhibition of the ERK phosphorylation sufficiently inhibited EGF-mediated Mcl-1 upregulation [37]. Sun et al. showed that the overexpression of ERK partly reversed EPOX-induced Mcl-1 degradation in tumor cells [32]. However, the detailed mechanism through which ERK affects Mcl-1 expression requires further investigation. In view of that (a) a decrease of Mcl-1 is essential for the induction of apoptosis by diverse apoptotic stimuli caused by different types of chemotherapeutic agents; (b) deactivation of ERK may result in Mcl-1 degradation; and (c) the administration of both HDACi and DHA synergistically regulate ERK phosphorylation and Mcl-1 expression, the combination treatment with HDACi and DHA should have a great clinical potential in the improvement of liver cancer treatment.

In light of the findings that (a) DHA induced more cell death in liver cancer cells bearing wild-type p53, (b) HDACi can lead to upregulation of p53, we rationally assumed that the combined treatment with DHA and HDACi increased apoptosis probably via inducing p53 expression in Hep G2 cells. However, more evidence should be obtained to verify the assumption. On the other hand, cells with p53 mutants have been demonstrated to be less sensitive to DHA, but can significantly respond to HDACi treatment [38]. Therefore, if the combination of both agents is used to treat p53-mutated cells, the apoptosis induced is likely to be comparable with that in cells with wild-type p53. In fact, such an assumption is proved in our present experiment. This finding indicates that HDACi and DHA in combination can be applied to both p53-wide type and p53-mutated liver cancer with similar efficacy. This is of clinical significance since p53 mutants are presented in most of liver cancer cases.

According to our results that (a) *in vitro* data showed that combination of HDACi and DHA significantly reduced cell viability in both cells, and (b) *in vivo* data revealed a remarkable decrease of Ki-67, this combined treatment resulted in significant cell growth inhibition which may lead to the resulting antitumor effects. However, further study should be carried on to disclose the exact mechanism through which cell growth inhibition, in addiction to apoptosis, caused by combination of HDACi and DHA contributed to tumor inhibition.

In conclusion, our *in vitro* and *in vivo* data highlight that the combination treatment with HDACi and DHA has a synergic effect in the induction of liver cancer cell death, and this strategy can also reduce the dose of HDACi and thus it may circumvent the inherent toxicity. Mechanically, our study has demonstrated that the combination treatment can regulate ERK phosphorylation and Mcl-1 expression to induce apoptosis of liver cancer cells independent of p53 status.
